# Serotyping, antibiogram, and detection of bacterial pathogens associated with bovine respiratory disease in selected areas of Ethiopia

**DOI:** 10.1186/s13620-022-00210-z

**Published:** 2022-03-03

**Authors:** Mirtneh Akalu, BhadraMurthy Vemulapati, Takele Abayneh, Teferi Degefa, Getaw Deresse, Esayas Gelaye

**Affiliations:** 1 Department of Biotechnology, Koneru Lakshmaiah Education Foundation, Vaddeswaram, Gunture, 522502 India; 2 National Veterinary Institute, P.O.Box: 19, Bishoftu, Oromia Ethiopia

**Keywords:** Antibiogram, Ethiopia, *H*, *somni*, *M. haemolytica*, *P. multocida*, Serotype

## Abstract

**Background:**

Bovine Respiratory Disease (BRD) is a multifactorial and economically important illness of cattle. The current study was designed to characterize the major bacterial pathogens associated with BRD and determine the antibiotic susceptibility patterns of isolates. Samples were collected from 400 pneumonic cases of cattle.

**Results:**

Laboratory assay revealed isolation of 376 (94.0%) bacterial pathogens. The most prevalent bacterial pathogens recovered were *Mannheimia haemolytica* (*M. haemolytica*) followed by *Pasteurella multocida* (*P. multocida*), *Histophilus somni* (*H. somni*), and *Bibersteinia trehalosi* (*B. trehalosi*) from 191 (50.80%), 81 (21.54%), 56 (14.89%), and 48 (12.77%) samples, respectively. *M. haemolytica* strains were confirmed using multiplex PCR assay through the amplification of *PHSSA* (~ 325 bp) and *Rpt2* (~ 1022 bp) genes. Capsular typing of *P. multocida* revealed amplification of serogroup A (*hyaD-hyaC*) gene (~ 1044 bp) and serogroup D (*dcbF*) gene (~ 657 bp). *B. trehalosi* isolates displayed amplification of the *sodA* gene (~ 144 bp). Besides, serotyping of *M. haemolytica* showed the distribution of serotype A:1 (82.20%), A:2 (10.47%), and A:6 (7.33%). Whereas, biotyping of *P. multocida* revealed a higher prevalence of biotype A:3 (83.95%), then A:1 (8.64%), A:2 (4.94%), and A:12 (2.47%). The majority of the retrieved isolates showed remarkable susceptibility to enrofloxacin, ciprofloxacin, sulfamethoxazole-trimethoprim, florfenicol, and ceftiofur (100%). Besides, varying degree of antimicrobial resistance was observed against streptomycin, gentamicin, penicillin-G, and ampicillin.

**Conclusions:**

The current findings confirmed that *M. haemolytica* (A:1) strain is the most common bacterial pathogen identified from BRD cases in the study areas of Ethiopia. Hence, continuous outbreak monitoring and evaluation of antibiotics susceptibility patterns of bacterial pathogens associated with BRD are indispensable to reduce the impact of BRD in the study areas. Further investigation of bacterial pathogens and genotypic analysis of pathogens from a wider area of the country is essential to design a cost-efficient control strategy.

## Introduction

Ethiopia is a home for various livestock species and is assumed to be among the leading countries in livestock population in Africa [1]. The total cattle population is estimated to be 65.3 million [2]. Despite the huge cattle population, the current productivity and commercialization of cattle remain very low due to diseases, inadequate feed, genetics of local breed, inefficient production system, and poor infrastructure along the value chain [3, 4]. The persistence of animal diseases such as Bovine respiratory disease (BRD) has continued to be a major constraint to the cattle population. It causes huge economic losses and reduced performance during and after the illness [5, 6]. Stresses, viral infections, nutritional, and environmental conditions are the predisposing factors that enhance the vulnerability of cattle to respiratory illness [7]. The bacterial pathogens that cause BRD include *M. haemolytica*, *P. multocida*, *H. somni*, *Mycoplasma* species, and *Trueperella Pyogens* (*T. pyogens*) [8, 9].

*M. haemolytica* and *B. trehalosi* strains were initially classified as *Pasteurella haemolytica* under the genus *Pasteurella*. Currently, these two pathogens are classified in two different genera of *Bibersteinia* and *Mannheimia* using DNA-DNA hybridization and 16S RNA [10]. The two biotypes are further classified into 17 serotypes. Thirteen serotypes classified to biotype A include (1, 2, 5, 6, 7, 8, 9, 11, 12, 13, 14, 16, and 17) and reclassified as *M. haemolytica*. On the other hand, four serotypes (3, 4, 10, and 15) are worth mentioned as *B. trehalosi* that belong to biotype T [11]. Serotype A:11 was later reclassified as *M. glucosidal* [12]. *P. multocida* strains are currently classified into five capsular types or serogroups (A, B, D, E, and F) based on capsular polysaccharide and into 16 Heddleston lipopolysaccharides (LPS) serovars using the gel diffusion precipitation assay [13, 14]. Besides, Polymerase chain reaction (PCR) assay [15] and the development of multiplex PCR assay used to determine each capsular serogroup [16]. Moreover, serogroups were classified into eight LPS (L1 – L8) genotypes [17].

Regardless of the considerable studies conducted over the past several years on BRD, it is still a serious concern to exert a huge economic impact on the cattle population [18]. Effective control of BRD likely requires a combination of more definitive diagnosis, efficacious vaccines, therapeutic intervention, and improved management practices [19]. Thus, BRD is one of the diseases that demand efficient control strategies. Previous reports showed the extent of respiratory disease losses, estimated to be higher in Ethiopia [20–22]. Furthermore, the emergence of multidrug-resistant bacterial pathogens associated with BRD is considered a potential threat to the cattle population [23]. Hence, these call for continuous outbreak monitoring, identification of bacterial pathogens diversity, and surveillance of antibiotic susceptibility. Therefore, the current study was designed to characterize and determine the antimicrobial susceptibility pattern of the major bacterial pathogens associated with BRD in Ethiopia.

## Results

### Clinical and pneumonic lung examination

**Fig. 1:**
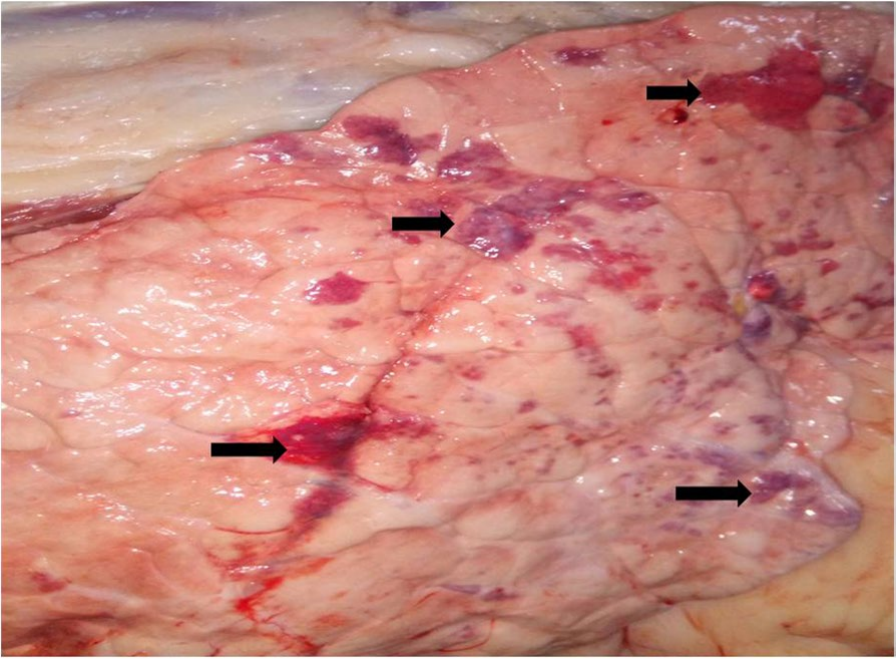
BRD infected lung of cattle showing pneumonic and hemorrhagic lesion (arrow)

Cattle suspected of respiratory infection were exhibited marked depression, loss of appetite (anorexia), severe respiratory distress, and pyrexia (high fever > 40.0 °C) which is commonly known as DART. Besides, coughing, salivation, lacrimation, and respiratory grunts were observed in advanced cases of the diseases. Cattle slaughtered at the abattoir were inspected for typical gross pathological lesions. Examined lung showed firm, friable, irregularity in shape, consolidation, and dark red color. In advanced cases, pulmonary parenchymal consolidation and interstitial edema were observed (Fig. 1).

### Bacterial isolation and distribution

A total of 400 cattle were examined in the present study. Bacteriological and PCR assay revealed the identification of 376 (94.0%) bacterial pathogens. Isolates were identified from 182 (91.0%) nasopharyngeal swab and 194 (97.0%) pneumonic lung tissue samples. The distribution of bacterial pathogens revealed a higher prevalence in adult cattle 220 (95.65%) than calves 156 (91.76%). Prevalence was slightly higher in female cattle 120 (94.49%) compared to male cattle 256 (93.77%). Besides, the prevalence in cross breeds 34 (94.44%) was higher than 342 (93.96%) local breeds. The highest prevalence was observed in poor body condition cattle 83 (97.65%) than moderate 109 (93.16%) and good body conditions 184 (92.93%). Table 1 shows the prevalence of the major bacterial pathogens associated with BRD. The current finding revealed that there was a significant difference (*P* < 0.05) between the prevalence of bacterial pathogens and age, sex, and body condition. However, there was no significant difference (*P* > 0.05) between the prevalence of pathogens and breed.

**Table 1:** Prevalence of the major bacterial pathogens associated with BRD

Variables		Samples				Total		Chi-square *P*-value
		Nasopharyngeal swab (*n* = 170)		Pneumonic lung tissue (*n* = 230)				
		*n*	Prevalence (%)	*n*	Prevalence (%)	*n* (%)	Prevalence (%)	
Age	Calves	125	111 (88.80)	45	45 (100)	170 (42.5)	156 (91.76)	0.00001
	Adult	75	71 (94.67)	155	149 (96.13)	230 (57.5)	220 (95.65)	
**Total**		**200**	**182 (91.0)**	**200**	**194 (97.0)**	**400**	**376 (94.0)**	
Sex	Male	86	72 (83.72)	187	184 (98.39)	273 (68.25)	256 (93.77)	0.00001
	Female	114	110 (96.49)	13	10 (76.92)	127 (31.75	120 (94.49)	
**Total**		**200**	**182 (91.0)**	**200**	**194 (97.0)**	**400**	**376 (94.0)**	
Breed	local	183	166 (90.71)	181	176 (97.24)	364 (91.0)	342 (93.96)	0.869
	Cross	17	16 (94.12)	19	18 (94.74)	36 (9.0)	34 (94.44)	
**Total**		**200**	**182 (91.0)**	**200**	**194 (97.0)**	**400**	**376 (94.0)**	
Body condition	poor	70	68 (97.14)	15	15 (100)	85 (21.25)	83 (97.65)	0.00001
	moderate	94	87 (92.55)	23	22 (95.65)	117 (29.25)	109 (93.16)	
	Good	36	27 (75.0)	162	157 (96.91)	198 (49.5)	184 (92.93)	
**Total**		**200**	**182 (91.0)**	**200**	**194 (97.0)**	**400**	**376 (94.0)**	

*n* – Sample size

The major bacterial pathogens encountered from suspected cases of BRD in the current study were shown in Table 2. The commonest bacterial pathogens encountered in the current study were *M. haemolytica*, *P. multocida*, *H. somni*, and *B. trehalosi* with the prevalence of 191 (50.80%), 81 (21.54%), 56 (14.89%), and 48 (12.77%), respectively. Out of these isolates, 111 (88.8%) were isolated from the nasopharyngeal swabs of calves and 71 (94.67%) from adult cattle. Besides, 45 (100%) and 149 (96.13%) were identified from pneumonic lung tissue of calves and adult cattle, respectively. There was no significant difference (*P* > 0.05) between the prevalence of pathogens and isolates type identified.

**Table 2:** The prevalence of the major bacterial pathogens from calves and adult cattle

Isolates	Calves		Adult cattle		Total	Chi-Square *P*-Value
	Nasopharyngeal swab (*n* = 125)	Pneumonic lung (*n* = 45)	Nasopharyngeal swab (*n* = 75)	Pneumonic lung (*n* = 155)		
*M. haemolytica*	68 (61.26)	18 (40.0)	35 (49.30)	70 (46.98)	191 (50.80)	.125*
*P. multocida*	21 (18.92)	10 (22.22)	13 (18.31)	37 (24.83)	81 (21.54)	
*B. trehalosi*	14 (12.61)	8 (17.78)	11 (15.49)	15 (10.07)	48 (12.77)	
*H. somni*	8 (7.21)	9 (20.0)	12 (16.90)	27 (18.12)	56 (14.89)	
Total	111 (88.8)	45 (100)	71 (94.67)	149 (96.13)	376 (94.0)	

*n -* Sample size, * the result is not significant at *P* < .05

### PCR assay

**Fig. 2:**
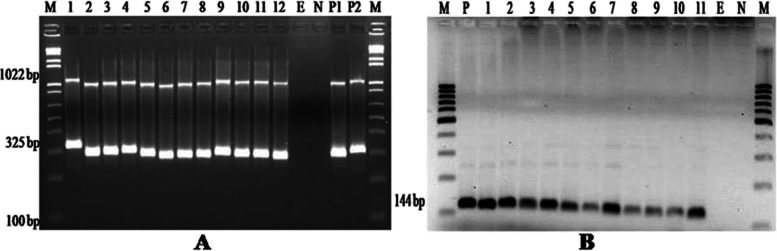
**A** Multiplex PCR amplification profile of PHSSA gene (~325 bp) and Rpt2 gene (~1022 bp) of M. haemolytica serotype1 specific strains. **B** B. trehalosi PCR

**Fig. 3:**
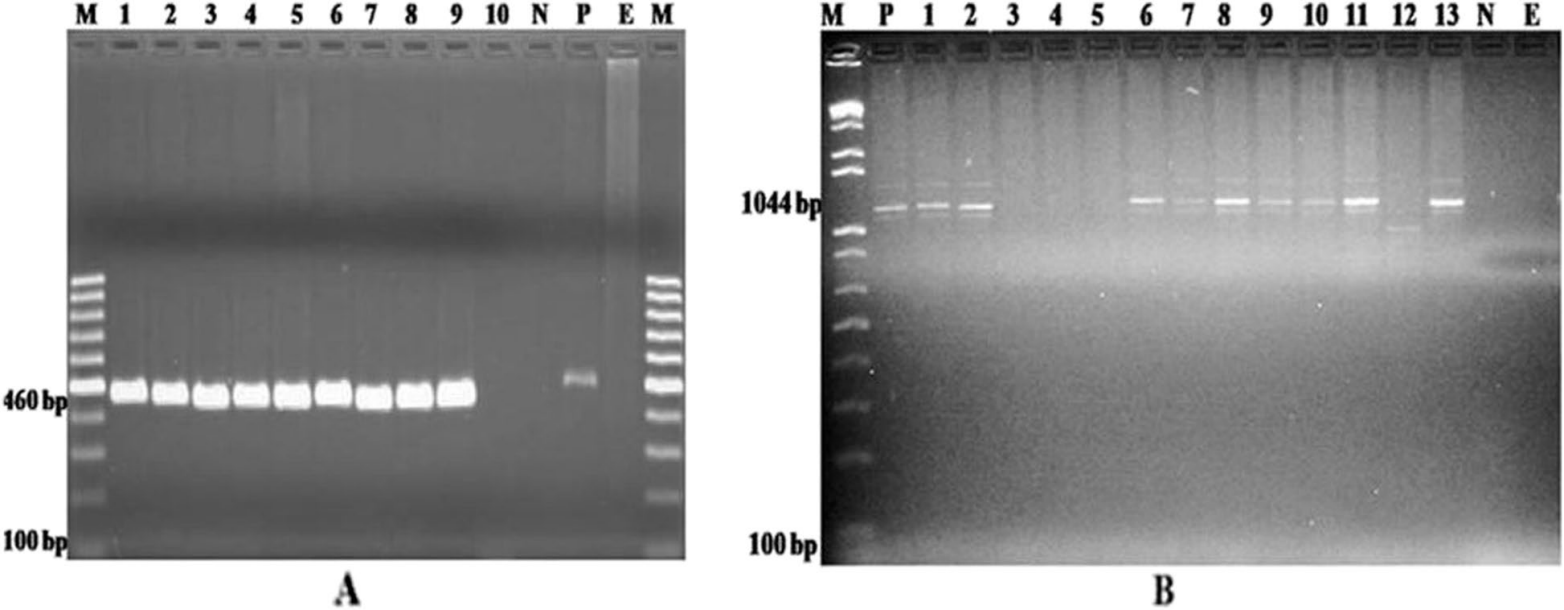
**A** PCR amplified product agarose-gel-electrophoresis of KMT1 gene (~460 bp) specific for P. multocida. **B** PCR amplified product agarose-gel-electrophoresis of

Multiplex PCR assay of *M. haemolytica* targeting *PHSSA* and *Rpt2* gene showed the desired amplification of band size at ~ 325 bp and ~ 1022 bp, respectively. While *B. trehalosi* isolates were found positive for the presence of the *sodA* gene by conventional PCR assay (Fig. 2A and B). *B. trehalosi* also revealed the desired band size of the *sodA* gene at ~ 144 bp. PCR assay of *P. multocida* revealed amplification of ~ 460 bp size for species-specific detection. *P. multocida* capsular typing confirmed by the presence of the *hyaD-hyaC* gene of serogroup A specific and amplified product showed band size of ~ 1044 bp (Fig. 3A and B).

### Serotypes and biovars

*M. haemolytica* serotyping revealed the identification of 157 (82.20%), 20 (10.47%), and 14 (7.33%) serotype A:1, A:2, and A:6, respectively. Isolates from nasopharyngeal swab samples showed serotype A:1 (73.79%), A:2 (18.48%), and A:6 (8.74%). Whereas, isolates from pneumonic lung tissue samples revealed serotype A:1 (92.05%), A:2 (2.27%), and A:6 (5.68%) as shown in Table 3. Further, characterization of *P. multocida* revealed the identification of *P. multocida* subspecies *multocida*. Thus, Ornithine decarboxylase (ODC) producing isolates belonged to biovar A:3, A:1, A:2, and A:12 from 78.69, 11.47, 6.56, and 3.28%, respectively.

**Table 3:** The distribution of *M. haemolytica* serotypes

Sample type	Isolates	Serotypes percentage			Chi-square *P*-value
		A:1	A:2	A:6	
Nasopharyngeal swab	103	76 (73.79%)	18 (17.48%)	9 (8.74%)	0.0015*
Pneumonic lung tissue	88	81 (92.05%)	2 (2.27%)	5 (5.68%)	
Total	191	157 (82.20%)	20 (10.47%)	14 (7.33%)	

* The result is significant at *P* < .05

### Antimicrobial susceptibility test (AST)

Tables 4 and 5 showed the antimicrobial susceptibility and multidrug-resistance patterns of the major bacterial pathogens against antimicrobial agents. Isolates were 100% susceptible to enrofloxacin, ciprofloxacin, ceftiofur, and florfenicol. Besides, susceptibility was observed in tetracycline (> 75%), and Oxytetracycline (> 80%). *M. haemolytica* showed varying degrees of multidrug-resistance against streptomycin, gentamicin, penicillin-G, and ampicillin 78.5, 72.8, 43.2, and 38.7%, respectively. *P. multocida* revealed multidrug resistance against streptomycin (84.0%), gentamicin (80.2%), Penicillin-G (48.1%), and Ampicillin (43.3%). *H. somni* exhibited multidrug resistance against streptomycin (71.4%), gentamicin (66.1%), Penicillin-G (51.8%), and Ampicillin (42.9%).

**Table 4:** Antimicrobial susceptibility assay of the major bacterial pathogens of BRD

Class	Antimicrobials	Disc conc.	Standard breakpoints	Susceptibility percentage of isolates (%)		
				M. H	P.M	H.S
β-lactam	Penicillin-G	10 U	R ≤ 28; S ≥ 29^a^	56.8	51.9	48.2
	Ampicillin	10 μg	R < 17; S ≥ 17^b^	61.3	56.7	57.1
Fluoroquinolones	Enrofloxacin	5 μg	R ≤ 16; S ≥ 21^a^	100	100	100
	Ciprofloxacin	5 μg	R < 27; S ≥ 27^b^	100	100	100
Aminoglycosides	Streptomycin	100 μg	R ≤ 11; S ≥ 15^a^	21.5	16.0	28.6
	Gentamycin	10 μg	R ≤ 12; S ≥ 15^a^	27.2	19.8	33.9
Tetracyclin**e**	Tetracycline	30 μg	R < 24; S ≥ 24^b^	81.7	77.8	87.5
	Oxytetracycline	30 μg	R ≤ 14; S ≥ 19^a^	85.3	83.9	91.1
	Sulfamethoxazole-trimethoprim	25 μg	R ≤ 10; S ≥ 16^a^	100	100	100
Phenicols	Florfenicol	30 μg	R ≤ 14; S ≥ 19^a^	100	100	100
Cephalosporin	Ceftiofur	30 μg	R ≤ 17; S ≥ 21^a^	100	100	100

M.H *M. haemolytica*, P.M *P. multocida***,** H.S *H. somni*., Conc. Concentration

^a^Clinical Laboratory Standard Institute (VET01S)

^b^The European Committee on Antimicrobial Susceptibility testing (EU vet-CAST)

**Table 5:** Multidrug-resistance patterns of the major bacterial pathogens associated with BRD

Class	Antimicrobial agents	Bacterial pathogens and resistance percentage (%)		
		*M. haemolytica*	*P. multocida*	*H. somni*
β-lactam	Penicillin-G	43.2	48.1	51.8
	Ampicillin	38.7	43.3	42.9
Aminoglycosides	Streptomycin	78.5	84.0	71.4
	Gentamycin	72.8	80.2	66.1
Tetracycline	Tetracycline	18.3	22.2	12.5
	Oxytetracycline	14.7	16.1	8.9

## Discussion

Differentiation of BRD based on visual and clinical examination is difficult. Hence, diagnosis has to be supported with the identification of the exact pathogens and AST to overcome the growing global concern of antimicrobial resistance. The finding in the present study revealed an overall incidence of 376 (94.0%) bacterial pathogens associated with BRD. Isolates were recovered from 182 (91.0%) nasopharyngeal swabs and 194 (97.0%) pneumonic lung tissue samples. The most prevalent bacterial pathogen recovered in this study was *M. haemolytica* strain 191 (50.80%) followed by *P. multocida* 81 (21.54%). Besides, *H. somni* and *B. trehalosi* were isolated from 56 (14.89%) and 48 (12.77%) pneumonic samples, respectively.

The current finding showed the potential impact of *M. haemolytica* and *P. multocida* in the study areas. The incidence of *M. haemolytica* was higher than previous reports of 29.2% [21], 10.13% [24], 10.67% [20], and 46.4% [22] which were identified from different parts of the country. The present study proves a higher incidence of *M. haemolytica* as compared to the other pathogens associated with BRD. Besides, *P. multocida* recovery from 81 (21.54%) pneumonic cases of cattle was higher than the previous reports of 3.34% [22] and 13.29% [20] but lower than 39.2% [21]. The occurrence of *B. trehalosi* from 48 (12.77%) cases was in agreement with 14.3% [22] and 12.67% [24]. Moreover, this study proves the presence of *H. somni* with an incidence rate of 56 (14.89%) in the study areas, which has been described only recently from cases of BRD in Ethiopia.

Isolation and differentiation of *M. haemolytica* and *B. trehalosi* isolates is difficult due to their phenotypic relatedness. Thus, the molecular assay was used as a confirmatory method in the current study. *M. haemolytica* virulence-associated genes are ideal targets for rapid molecular characterization due to *M. haemolytica* genomic fragments homologous to *PHSSA* have been identified from many strains of the isolate [25]. *PHSSA* represents *M. haemolytica* virulence-associated (species-specific) genes [26] and *Rpt2* (species-specific) locus in *M. haemolytica* has a possible role in modulation of type III restriction-modification system [27]. Multiplex PCR assay of *M. haemolytica* revealed simultaneous amplification of the two gene fragments (*PHSSA* and *Rpt2*). Hence, PCR assay targeting *PHSSA* and *Rpt2* genes become an appropriate molecular diagnostic tool with a high degree of discriminating efficiency. In the present study majority of *M. haemolytica* strains displayed the desired amplification band size of *PHSSA* (~ 325 bp) and *Rpt2* (~ 1022 bp) genes. Moreover, presumptively identified *B. trehalosi* PCR products showed amplification of the *sodA* gene (~ 144 bp) coding manganese-dependent superoxide dismutase.

Capsular typing of *P. multocida* targeting *hyaD-hyaC* gene (~ 1044 bp) confirmed the preponderance of serogroup A strains in the present study. Capsular type A was recovered from 76 (93.83%) isolates and capsular type D was identified from 5 (6.17%) isolates. The current finding is in agreement with the report of 93.7% capsular type A and 6.3% capsular type D [28] Moreover, previous study reported that serogroup A is the most prevalent isolate from cattle [29]. Further serotyping analysis of 191 *M. haemolytica* isolates revealed that 157 (82.20%), 20 (10.47%), and 14 (7.33%) isolates were classified to serotype A:1, A:2, and A:6, respectively. Thus, serotype A:1 was considered as the prevalent pathogen to cause BRD in the study areas. Likewise, Ornithine decarboxylase (ODC) producing *P. multocida* isolates categorized to biotype A:3 (78.69%), followed by biotype A:1 (11.47%), A:2 (6.56%), and A:12 (3.28%). Thus, *P. multocida* A:3 strain was considered among the principal respiratory pathogens in cattle.

Antimicrobial resistance is a growing global threat that calls for appropriate use and antimicrobial choice during treatment. In this study, the antibiotics susceptibility pattern of *M. haemolytica*, *P. multocida*, and *H. somni* strains were exhibited remarkable susceptibility to enrofloxacin, ciprofloxacin, ceftiofur, and florfenicol (100% for each), tetracycline (> 75%), and Oxytetracycline (> 80%). However, *M. haemolytica* showed varying degrees of multidrug resistance against streptomycin (78.5%), gentamicin (72.8%), penicillin-G (43.2%), and ampicillin (38.7%). *P. multocida* revealed multidrug resistance against streptomycin (84.0%), gentamicin (80.2%), Penicillin-G (48.1%), and Ampicillin (43.3%). In addition, *H. somni* exhibited multidrug resistance against streptomycin (71.4%), gentamicin (66.1%), Penicillin-G (51.8%), and Ampicillin (42.9%). The current antibiotics susceptibility pattern-finding was supported by a few studies in Ethiopia [22]. However, the finding suggests further studies to investigate the contributing factors associated with multidrug-resistance and measure the association between antimicrobials use and exposure to BRD in the country.

In this study, the findings showed remarkable evidence of the major bacterial pathogens associated with BRD and their antibiotic susceptibility pattern. *M. haemolytica* (A:1) strain is the most predominant bacterial pathogen followed by *P. multocida* (A:3) strain to cause BRD. These two pathogens were considered as the principal bacterial pathogens associated with BRD infection in the study areas of Ethiopia. Besides, *B. trehalosi* and *H. somni* were associated with few pneumonic cases and perhaps considered as potential pathogens to cause significant impact in the study areas. However, the current finding lacks to show the potential impact of mycoplasma species and other emerging bacterial pathogens associated with BRD. Hence, these bacterial pathogens could be further investigated to know more about the current epidemiological scenario in Ethiopia. Such studies could help in designing efficient prevention and control strategies.

## Conclusion

The current finding described the major bacterial pathogens prevalence, serotypes, and antibiotics susceptibility pattern. The phenotypic and molecular assay confirmed that *M. haemolytica* (A:1) is the most common bacterial pathogen identified from BRD cases in the study areas of Ethiopia. Besides, continuous outbreak monitoring and surveillance of antimicrobial susceptibility is indispensable to decide on the drug of choice attributable to the development of multidrug-resistant strains. Therefore, the current findings suggest further comprehensive studies to investigate strain distribution, the antigenic relationship among strains to understand the molecular epidemiology, and other bacterial pathogens associated with BRD at the national level to design a cost-efficient control strategy.

## Methods

### Study area and animal

Samples were collected from different agro-ecological zones of Ethiopia (Bale-Robe located at 7°7′N, 40°0′E, Asosa situated at 10°04′N, 34°31′E, Bishoftu found at 8°45′N, 38°59′E), Yabelo located 4°53′N, 38°5′E), and Mekele set at 13°29′N, 39°28′E). These areas were located from 550 to 2492 m above sea level (m.a.s.l). Samples were collected from a total of 400 BRD suspected cases. Laboratory analysis was carried out at the National Veterinary Institute (NVI), Ethiopia.

Samples were collected from study animals based on respiratory clinical signs and postmortem examination associated with age, sex, breed, and body conditions. Clinical signs of bacterial pneumonia in active cases were evaluated for combination of signs including depression and fever (39 °C - 40 °C), serous to mucopurulent nasal discharge, moist cough, and a rapid and shallow respiratory rate are the classic components of a case definition for early BRD cases. Sever cases characterized for pleurisy, irregular breathing pattern, grunting, and unthrift appearance associated with pulmonary abscesses.

### Sample collection and sample size

A cross-sectional study with a purposive sampling method was employed to collect samples. Samples were collected from suspected BRD cases brought to veterinary clinics and an abattoir survey was conducted to collect pneumonic lung tissue samples. Since there is no similar research study in the area, the expected prevalence was assumed 50%. The sample size for the study was calculated using a 95% confidence level and required 5% precision [30].

$$\mathrm{N}=\frac{(1.96)^2{P}_{\mathrm{exp}}\left(1-{P}_{\mathrm{exp}}\right)}{{\left(\mathrm{d}\right)}^2}$$

Where *N* is the required sample size,

*P*_exp_ is expected prevalence, and

*d* is required precision.

The required sample size was calculated to be 384, but 5% samples were considered for the precision of sampling from the study areas and this makes the total samples to be 400.

### Nasopharyngeal swab

Respiratory cases of cattle were examined using the Wisconsin clinical respiratory scoring method from 0 (normal) to 3 (severe) cases. The scoring was made by assessing the five clinical signs (fever, lacrimation, nasal discharge, coughing, and ear position). Cattle with higher clinical respiratory scoring (≥ 5) were considered for sampling. Nasopharyngeal swab samples from the nasopharynx were collected from suspected cattle. The sample was collected using a laryngeal swab (MWE dry swab, England). Briefly, the external nares was cleaned with a dry paper towel for any frank discharge or detritus from the nares. A sterile plastic swab was directed via the ventral nasal meatus into the nasopharynx, rotated vigorously against the pharyngeal mucosa for 30 – 45 s at the contra-lateral side. The swab was retracted by taking care not to touch the nares and samples were placed into a sterile screw-capped test tube with a modified Cary-Blair Medium (Park Scientific, UK).

### Pneumonic lung

Pneumonic lung tissue samples were inspected and evaluated grossly. A small portion of the lung tissue (~ 3 × 3 mm) sample was aseptically taken from the edge of the lesion. Samples were collected immediately after slaughter and kept in a sterile screw-capped universal bottle. Samples were transported and maintained in a cold chain.

### Bacteriological assay

Nasopharyngeal samples were inoculated comparably onto MacConkey and blood agar base (HiMedia, India) supplemented with 5% sheep blood. Lung samples were processed (minced, vortex, centrifuged (3200 x g, 3 min), supernatant discarded, sediment reconstituted) and the suspension was cultured onto MacConkey and blood agar. The remaining lung tissue suspension was stored in 20% glycerol at − 80 °C and later processed to isolate *H. somni*. Ten microliter of the frozen stock was inoculated onto blood agar (37 °C for 48 h in 5-10% CO_2_). Presumptive colonies of the major bacterial pathogens were identified based on the standard morphological, cultural, and biochemical assay.

### Biochemical assay

Isolates were further analyzed for Grams staining, oxidase, catalase, ornithine decarboxylase (ODC) reaction, indole production, urease, and nitrate reduction. Identification of bacteria pathogens to species level was carried out based on sugar fermentation reaction (glucose, sucrose, lactose, arabinose, trehalose, dulcitol, mannitol, sorbitol, and D-xylose).

### Serotyping of *M. haemolytica*

Rapid plate agglutination assay was employed to characterize the serotypes of *M. haemolytica* strains as described previously [23]. Rabbit antisera were prepared against reference strains of *M. haemolytica* which were kindly provided by the NVI, Ethiopia.

### Molecular Characterization

#### DNA extraction

Genomic DNA was extracted using DNeasy® Blood and Tissue kit (QIAGEN GmbH, Germany) following the manufacturer’s instructions.

#### Multiplex PCR assay of *M. haemolytica*

*M. haemolytica* serotype-specific virulence-associated (*PHSSA*) genes and methyltransferase coding (*Rpt2*) gene were used in multiplex PCR assay as described in prior studies [25]. The oligonucleotide sequences used in this study was illustrated in Table 6. PCR assay was conducted in a final volume of 25 μl reaction mixture containing IQ supermix (10 μl of Bio-Rad, USA), primer pair (2 μl of 5 pm/μl), RNase free water (3 μl), and template DNA (3 μl). Alpha thermal cycler (PCR max, Ac 296, UK) was used for amplification (Table 7).

**Table 6:** Isolates and target gene sequences (5′ to 3′) used in PCR assay

Isolates	Gene	Primers	Sequence (5′ to 3′)	Size (bp)	Reference
*M. haemolytica*	*PHSSA*	PHSSA (F)	TTC ACA TCT TCA TCC TC	325	[25]
		PHSSA (R)	TTT TCA TCC TCT TCG TC		
	*Rpt2*	Rpt2 (F)	GTT TGT AAG ATA TCC CAT TT	1022	
		Rpt2 (R)	CGT TTT CCA CTT GCG TGA		
*P. multocida*	KMT1	KMT1T7 (F)	ATC CGC TAT TTA CCC AGT GG	460	[15, 26]
		KMT1SP6(R)	GCT GTA AAC GAA CTC GCC AC		
Serogroup A	*hyaD-hyaC*	capA (F)	TGC CAA AAT CGC AGT CAG	1044	[16]
		capA (R)	TTG CCA TCA TTG TCA GTG		
Serogroup B	*bcbD*	capB (F)	CAT TTA TCC AAG CTC CAC C	760	[16]
		capB (R)	GCC CGA GAG TTT CAA TCC		
Serogroup D	*dcbF*	capD (F)	TTA CAA AAG AAA GAC TAG GAG CCC	657	[16]
		capD (R)	CAT CTA CCC ACT CAA CCA TAT CAG		
Serogroup E	*ecbJ*	capE (F)	TCC GCA GAA AAT TAT TGA CTC	511	[16]
		capE (R)	GCT TGC TGC TTG ATT TTG TC		
*B. trehalosi*	*sodA*	BtsodA (F)	GCC TGC GGA CAA ACG TGT TG	144	[31]
		BtsodA (R)	TTT CAA CAG AAC CAA AAT CAC GAA TG		

F *forward primer*, *R* reverse primer, *bp* base pair

**Table 7:** PCR assay reaction of the current isolates

Isolates	PCR reaction				Final extension
	Initial denaturation	Denaturation (35 cycles)	Annealing	Extension	
*M. haemolytica*	95 °C; 3 min	95 °C; 1 min	48 °C; 1 min	72 °C; 1 min	72 °C; 5 min
*P. multocida*	95 °C; 5 min	95 °C; 1 min	55 °C; 1 min	72 °C; 1.5 min	72 °C; 7 min.
*B. trehalosi*	95 °C; 5 min	95 °C; 30 s	55 °C; 30 s	72 °C; 40 s	72 °C; 5 min.

#### *PCR assay of P. multocida*

Species-specific primers were used in *P. multocida* PCR assay based on a previous report [26]. A reaction mix of 20 μl containing IQ supermix (10 μl), primer pair (2 μl of 5 pmol), RNase free water (3 μl), and DNA template (3 μl) was used in the PCR assay. Capsular typing was assayed using serogroup-specific (A, B, D, and E) primers. PCR assay was conducted in a final volume of 40 μl reaction mixture containing IQ supermix (20 μl), primer pair (6 μl of 5 pm/μl), RNase free water (2 μl), and template DNA (6 μl).

#### PCR detection of *B. trehalosi*

Specific primer targeting *sodA* gene coding for manganese-dependent superoxide dismutase was used to detect *B. trehalosi* as described in a previous study [31].

### Electrophoresis

Electrophoresis was carried out in agarose gel (2%). PCR product (10 μl) was mixed with a 6x loading buffer. One hundred bp or 1 kb plus DNA molecular marker (10 μl) was added into the first and last lane and run at 120 V for 60 min. The expected band size of PCR products was visualized under a gel documentation system (Uvitec, UK).

### AST

AST was conducted using the Kirby-Bauer disk diffusion method to evaluate the sensitivity pattern of the commonly used antimicrobials in the treatment of BRD. Antibiogram of the major bacterial pathogens was carried out against Enrofloxacin (ENR 5 μg), Ciprofloxacin (CIP 5 μg), Penicillin-G (P 10 units), Ampicillin (AMP 10 μg), Streptomycin (S 25 μg), Gentamicin (CN 10 μg), Tetracycline (TE 30 μg), Oxytetracycline (OT 30 μg), Sulfamethoxazole-trimethoprim (SXT 25 μg), Florfenicol (FFC 30 μg), and Ceftiofur (EFT 30 μg). The result was interpreted as described by the Clinical and Laboratory Standards Institute (CLSI) [32] and European committee on antimicrobial susceptibility testing EUvet-CAST [33].

### Analysis

Data were coded and stored in an excel spreadsheet. Descriptive statistics and Chi-square test was used for the analysis using STATA (STATA software version 11.0). Statistical significant level was considered at *P* < .05.

## Data Availability

All data supporting the findings of this study can be obtained from the corresponding author upon formal request.

## Abbreviations

AST: Antimicrobial susceptibility test
BRD: Bovine respiratory disease
DNA: Deoxyribose nucleic acid
NVI: National Veterinary Institute
PCR: Polymerase chain reaction
